# Bone Mineral Density in Severely Obese Women: Health Risk and Health Protective Risk Factors in Three Different Bone Sites

**DOI:** 10.3390/ijerph17197017

**Published:** 2020-09-25

**Authors:** Camila Kellen de Souza Cardoso, Maria do Rosário Gondim Peixoto, Ana Paula dos Santos Rodrigues, Carolina Rodrigues Mendonça, Cesar de Oliveira, Erika Aparecida Silveira

**Affiliations:** 1 Postgraduate Program in Health Sciences, Pontifical Catholic University of Goias, Goiânia 74605-020, Goiás, Brazil; camilacardoso_nut@hotmail.com; 2 Postgraduate Program in Nutrition and Health, Faculty of Nutrition, Federal University of Goias, Goiânia 74605-220, Goiás, Brazil; mrg.peixoto@uol.com.br; 3 Postgraduate Program in Health Sciences, Faculty of Medicine, Federal University of Goias, Goiânia 74605-220, Goiás, Brazil; anapsr@gmail.com (A.P.d.S.R.); carol_mendonca85@hotmail.com (C.R.M.); 4 Department of Epidemiology & Public Health, Institute of Epidemiology & Health Care, University College London, London WC1E 6BT, UK; c.oliveira@ucl.ac.uk

**Keywords:** bone tissue, bone health, morbid obesity, vitamin D, smoking

## Abstract

Factors associated with bone mineral density (BMD) are poorly known in severely obese individuals i.e., a body mass index (BMI) > 35 kg/m^2^. The objectives of this study were to describe the bone health profile of severely obese Brazilian women, to identify the health risk and health protective factors for BMD in this group and to assess whether these factors vary according to three different bone sites. BMD was assessed using dual-energy X-ray absorptiometry (DXA). This study analyzed baseline data from 104 women who had an average BMI of 43.7 ± 4.5 kg/m^2^ and presented the following BMD status: 1.283 ± 0.094 g/cm^2^ for total body, 1.062 ± 0.159 g/cm^2^ for vertebral column and 1.195 ± 0.134 g/cm^2^ for hip. They took part in the “Effect of nutritional intervention and olive oil in severe obesity” randomized clinical trial (DieTBra Trial). The risk factors negatively associated with lower BMD were age ≥50 years for the three bone sites i.e., total body, vertebral column and hip. Smoking for total body BMD (*p* = 0.045); BMI ≥ 50kg/m^2^ for vertebral column and hip; menopause for hip; high C-reactive protein (CRP) levels (*p* = 0.049), insufficient zinc (*p* = 0.010) and previous fracture for vertebral column (*p* = 0.007). The protective factors positively associated with BMD were physical activity (≥150 min/week (*p* = 0.001)) for hip; type 2 diabetes mellitus (DM2) (*p* < 0.0001) total body and adequate vitamin D levels from food consumption (*p* = 0.039) for vertebral column. A BMI ≥ 50 kg/m^2^ was a risk factor for lower BMD. The findings showed that protective and risk factors varied by bone site. The original study is registered with ClinicalTrials.gov. (protocol number: NCT02463435).

## 1. Introduction

Obesity is a public health concern with an increasing prevalence globally [1] with serious health consequences including poorer bone health [2,3]. Reductions in bone mineral density (BMD) cause diseases such as osteopenia and osteoporosis that directly affect the health of adults and, especially older adults [4]. Although there are several risk factors associated to BMD reduction, North-American data showed that women and people of older age are the most common ones [5,6]. However, little is known about bone health and associated factors in women with severe obesity as well as whether the level of obesity can affect their BMD.

Few studies showed beneficial effects of obesity on bone health. There are reasons to believe that these effects of obesity on BMD are context-dependent, since, in general, excess body fat exerts mechanical overload on the bones, in addition to hyperinsulinemia and hyperamylinemia that may negatively affect the bones [7,8,9]. A study revealed that a body mass index (BMI) greater than 40 kg/m^2^ had a beneficial effect on BMD [10]. However, there was a large dispersion of absolute values around the mean, indicating that this effect is not guaranteed for all morbidly obese individuals. Therefore, the positive effects of obesity on BMD do not alleviate its harmful effects on bone markers, like the BMD [2,3,10].

The relationship between obesity and bone metabolism is known to be complex and dependent on mechanical and biochemical factors. The mechanisms by which obesity adversely affects bone health involve dysfunction of bone regulating hormones and low-grade systemic inflammation in a context that may affect bone cell metabolism [2,3,9,11,12]. Although some studies support the positive effect of obesity on bone health, this might be related to the bone site. Considering the metabolic complexity and comorbidities of obesity, these individuals may be at higher risk for bone frailty depending on the bone site [2,3,9,10,13].

Therefore, because the evidence on the effect of severe obesity on BMD is still controversial and scarce and considering the obesity pandemic, in particular, the increase in incidence of BMI ≥35 kg/m^2^ in the last years [1], the present study represents an important contribution in the fields of obesity, nutrition and bone health. The main objectives of this study were (1) to describe the bone health profile of severely obese Brazilian women, (2) to identify the health risk and health protective factors for BMD in this group and (3) to assess whether these factors vary according to three different bone sites.

## 2. Methods 

### 2.1. Design and Study Participants

The data for this study are part of the baseline of a major randomized clinical trial called “Effect of nutritional intervention and olive oil in severe obesity-DieTBra Trial” [14,15,16,17,18,19,20,21,22]; details of the study design and subject recruitment and randomization are described in the literature [14,15,16,17,18,19,20,21,22]. Data collection was carried out at the Clinical Research Unit of the Faculty of Medicine Clinics Hospital, Federal University of Goiás, Brazil, between June 2015 and February 2016 [14,15,16,17,18,19,20,21,22]. The following eligibility criteria were adopted: women, aged between 18 and 64 years with a BMI ≥ 35 kg/m^2^ and weight ≤ 130 kg. Individuals with metal in their body such as rods and pins, post-bariatric surgery, weight loss > 8% in the last three months, previous nutritional treatment, pregnant, infants and with some type of disability were excluded. 

### 2.2. Ethical Aspects and RCT Registration

The DieTBra Trial was approved by the Research Ethics Committee of the Clinics Hospital of the Federal University of Goiás under protocol number 747.792/2014. All participants signed a written informed consent form. The major study was registered at the ClinicalTrials.gov. platform (NCT02463435). 

### 2.3. Sociodemographic, Lifestyle and Medication Data

The sociodemographic characteristics were sex, age, self-reported skin color, level of education and socioeconomic classification according to the Brazilian Association of Research Companies [23] that considers consumer goods, purchasing power, education, access to treated water and sewage. The lifestyle variables included smoking (smoker, ex-smoker, never smoked) [24], sun exposure (yes/no and time) and level of physical activity (PA). Alcohol consumption was evaluated by episodes of binge drinking on one occasion (≥5 and ≥4 doses for men and women, respectively) [25]. PA was evaluated using the “triaxial accelerometer ActiGraph wGT3X (ActiGraph, Pensacola, FL, USA)” positioned at the back of the non-dominant wrist and analyzed by ActiLife 6 software (ActiGraph, Pensacola, FL, USA). Each individual was instructed to use it 24 h a day for six consecutive days. The level of PA was categorized according to the recommended practice of ≥150 min/week of moderate to vigorous aerobic physical activity (MVPA) [26]. The outcome measures used in the present study were MVPA defined as estimated time spent in ≥10 min per bout during a week [27]. The continued use of the classes of drugs that reduce BMD was investigated, including glucocorticoids, proton pump inhibitors, anticonvulsants/neuroleptics, medroxyprogesterone acetate (MPA), aromatase inhibitors, gonadotrophin-releasing hormone (GnRH) agonists, thiazolidinediones/glitazones, calcineurin inhibitors, heparin and warfarin/anticoagulant, thyroxine/thyroid hormone, and loop diuretics [28,29].

### 2.4. Biochemical Tests and Health Conditions

The biochemical tests, methods and normality values are described in Table 1. Samples were collected after a 12-h fast period. The presence of type 2 diabetes mellitus (DM2) was assessed based on the use of hypoglycemic agents and/or fasting glucose ≥126 mg/dL and glycosylated hemoglobin ≥ 6.5% tests [30]. Hypothyroidism was investigated based on the use of thyroxine-based drugs and/or TSH > 4.12 mUI/L and free T4 < 0.7 ng/dL [31]. Menopause was assessed based on prior medical diagnosis based on responses to the question "*Has your doctor ever told you that you are in menopause?”*, if the answer was yes, then it was asked *“Do you take any menopause medication?”.* The presence of previous fractures or osteoporosis or osteopenia was investigated by the question *"Have you ever had a fracture?"* and “*Has your doctor ever told you that you have osteoporosis or osteopenia?*”

### 2.5. Calcium and Vitamin D Intake and Anthropometry

Calcium and vitamin D intakes were assessed by the average of three 24-Hour Dietary Recall (24 HR) evaluations [40]. Sufficient intake was defined according to the estimated average requirements (EARs) of ≥800 mg/day ≥ 10 mcg/day for calcium and vitamin D, respectively [41]. To evaluate the degree of obesity, the current weight was measured using a Welmy digital scale, with capacity of 300 kg and precision of 100 g. Height was measured using a stadiometer coupled to the digital scale, with precision of 0.1 cm [42]. BMI was then calculated and classified according to WHO guidelines [43].

### 2.6. Bone Densitometry

BMD was assessed by dual-energy X-ray absorptiometry (DXA) using a *GE Healthcare* bone densitometer (Lunar DPX NT, 130 kg capacity and 1.03 m width). To perform the DXA, each patient was placed in dorsal decubitus with the arms close to the body. If it was not possible to scan both arms under the DXA due to the patient’s size, the left arm was excluded from the analysis while the right arm was analyzed and duplicated [44]. The total body, total vertebral column, and total hip BMD were measured in g/cm^2^.

### 2.7. Statistical Analysis

A database was built using EPI DATA^®^ version 3.1 (Statacorp LP, College Station, TX, USA), with double entry typing for validation and consistency analysis. Statistical analyses were performed using Stata/SE 13.0. (Statacorp LP, College Station, TX, USA). The absolute and relative frequencies, means, and standard deviations were calculated. The Kolmogorov-Smirnov test was used to verify the normality of the data. Bivariate analysis was performed using Student’s *t*-test or analysis of variance (ANOVA), followed by Bonferroni for comparison of means based on a level of significance of 5%. The outcomes were total body, total vertebral column and total hip BMD, which were analyzed as continuous values.

Variables with a *p* value < 0.20 in the simple linear regression analysis were included in multiple linear regression (MLR) analysis according to the hierarchical analysis model and were included in the final model only if *p* < 0.05. The hierarchical levels of MLR were as follows: first level-sociodemographic variables (age, skin colour, social class and education); second level-clinical and lifestyle variables (smoking, binge drinking, physical activities ≥150 min/week, previous fracture, menopause, diabetes, hypothyroidism, use of medications, BMI, calcium and vitamin D intake); and third level-biochemical tests (C-reactive protein (CRP), zinc, parathyroid hormone (PTH), thyroid stimulating hormone (TSH) and fasting glycemia). The explanatory variables were used in the categorized form (dummy variables) to verify groups of individuals with higher or lower BMD in the adjusted analysis. After the final model, quality analysis was carried out by means of residual graphs; multicollinearity was also verified using variation inflation factor (VIF).

## 3. Results 

The study included 104 severely obese women, with mean age of 40.2 ± 8.5 years (range: 21 to 62 years), average weight of 109.7 ± 11.5 kg (range: 79.6 to 129 kg) e average height of 1.59 ± 0.6 m (range: 1.45 to 1.73 m) and mean BMI of 43.7 ± 4.5 kg/m^2^ (35.0 to 54.8 kg/m^2^). Most had white or brown skin color (84.6%), with ≥9 years of education (67.3%), and belonged to the economic class A–C (82.7%). The most frequent BMI category was 40 to 49.9 kg/m^2^ (70.2%) (Table 2). Mean BMD at the three bone sites studied were 1.283 ± 0.094 g/cm^2^ for total body, 1.062 ± 0.159 g/cm^2^ vertebral column and 1.195 ± 0.134 g/cm^2^ for hip.

Women aged ≥50 years had significantly lower BMD than those aged 18–49 years at the hip and vertebral column bone sites. Total body BMD was greater in older women (40–49 years) than in younger (18–39 years) (*p* < 0.001). Total body BMD was lower in smokers/ ex-smokers than in those who never smoked (*p* = 0.043) (Table 2). 

Regarding lifestyle and clinical variables, menopause was associated with lower BMD at the three bone sites (*p* = 0.025, 0.025, and <0.001, respectively). BMI ≥ 50 kg/m^2^ was associated with lower vertebral column BMD compared to BMI 35–39.9 kg/m^2^ (*p* = 0.046), same to previous fracture (*p* = 0.005). Patients with diabetes had higher total body BMD compared to non-diabetic (*p* = 0.039) (Table 2).

Elevated CRP level (*p* = 0.040) and lower serum zinc (*p* = 0.016) were associated with lower total vertebral column BMD. Adequate serum levels of vitamin D (*p* = 0.043) were associated with higher total hip BMD (Table 3).

Based on simple linear regressions, the variables included in the MLR for total body BMD were age, smoking, previous fracture, menopause, diabetes, hypothyroidism and medications. For total vertebral column BMD, the following variables were included: age, skin color, education, smoking, MVPA, previous fracture, menopause, BMI, vitamin D intake, serum zinc, PTH and CRP. Finally, for total hip BMD, variables included were age, smoking, excessive alcohol consumption, MVPA, previous fracture, menopause, BMI, calcium intake, serum vitamin D, serum zinc, TSH, and CRP (Table 4). 

After MLR adjustments, age ≥ 50 years was associated with lower BMD at three sites: total body (*p* < 0.001), vertebral column (*p* < 0.001) and hip (*p* = 0.001). Smokers/ex-smokers had lower total body BMD (*p* = 0.045). DM2 (*p* > 0.001) was associated with higher total body BMD. Risk factors associated with lower total vertebral column BMD were previous fracture (*p* = 0.007), greater BMI obesity (≥50 kg/m^2^) (*p* = 0.022), vitamin D insufficient intake (*p* = 0.039), elevated CRP levels (*p* = 0.049) and insufficient serum zinc (*p* = 0.010). Risk factors for lower hip BMD were menopause (*p* = 0.001) and higher BMI (≥50 kg/m^2^) (*p* = 0.045). In addition, ≥150 min/week of MVPA was associated with higher total hip BMD (*p* = 0.001) (Table 5).

## 4. Discussion 

In this study, we identified health risk and protective factors for BMD in three bone sites in women with severe obesity. Our main findings contribute to the knowledge in this field and may lead to preventive and clinical strategies. In addition, we described the bone health profile of severely obese women and assessed whether risk and protective factors differ in the three bone sites investigated. It is important to highlight that severe obesity is the fastest growing obesity group in recent years [1] and due to its particular epidemiological and clinical traits; the development of research focusing on this target population is increasingly relevant. Our results indicate that there are subgroups among severely obese women (BMI ≥ 35 kg/m^2^) with different levels of bone mass, depending on the evaluated bone site.

Negative association between BMD and age ≥50 years was observed at the three evaluated bone sites. The sex-age binomial is a classic determinant of BMD since the frequency of osteoporosis is three times higher in women and the prevalence of hip fractures is higher after 50 years [5,45]. In aging, bone reabsorption is normal, but there is a gradual reduction in bone formation capacity, resulting in bone demineralization over the years [46,47]. In addition, obesity is a concern for bone health during ageing because obesity damages bone health over time due to multiple factors that may affect bone cell metabolism [3].

Estrogen reduction is related to accelerated bone loss in women, which occurs at menopause, because this hormone has a significant anti-bone resorption effect [48,49]. This is consistent with our study in which menopause was associated with lower total hip BMD. It is worth mentioning that one of the main reasons for monitoring osteoporosis in this period is the prevention of bone fragility, so identifying women with the lowest BMD is a clinical priority. Low BMD, particularly in the hip, is a strong risk factor for fracture, being estimated that each 1-SD reduction in BMD increases the risk of fracture by 2 to 3 times [50].

Previous fracture was associated with lower total vertebral column BMD in severely obese women, similarly to findings from other study with middle-aged women [51]. Previous fracture, especially in adult life, along with low bone mass leads to a vicious cycle since the fracture aggravates the appearance of osteopenia or osteoporosis and vice versa, of particular concern for the posterior risk of osteoporosis [51,52]. A study in obese women with fractures showed that about 23% had previous fractures. The authors of this study concluded that in obese individuals, there is an increased risk of falls and lower physical mobility, that are important etiological factors for fractures [53].

Smokers had lower total body BMD, which corroborates a study that found lower femur and vertebral BMD in female smokers compared to non-smokers [54]. Nicotine, present in tobacco, exerts a toxic action on osteoclasts and osteoblasts, and smoking may have an anti-estrogen effect, since female smokers had lower estrogen concentration and higher rate of early menopause [55]. Tobacco can affect vitamin D metabolism because serum levels of this vitamin are significantly lower in smokers. Therefore, intestinal calcium absorption is reduced, resulting in impaired bone mineralization [55,56]. Smokers have a 300% higher chance (OR 4.0, CI 95% 1.05–15.5) of having osteoporosis compared to non-smokers. [57].

In the present study, PA was associated with higher hip BMD in severely obese women. There are several benefits of regular PA practice that are well documented in the literature [26] and corroborate our findings. Our results are consistent with a cohort of adults and elderly who showed that increased PA level was associated with increased total hip BMD [58]. Like muscle, bone is a tissue that responds to PA by stimulating osteogenesis in order to develop denser bones to withstand the impact of exercise and the force of gravity. In addition, PA favors the increase in muscle mass that affects the dynamic mechanical overload on the bones, inducing bone formation [58,59].

Increased total body BMD was associated with DM2 in severely obese women in the present study. It was an intriguing finding because endocrine diseases such as DM2 seem to be a risk factor for lower BMD [60]. However, no previous similar result was found in this population. A cohort of adults and older adults reported higher femoral neck and lumbar vertebral column BMD among hyperglycemic patients with DM2 [61]. Despite that, these individuals had a relative fracture risk of 1.62 (95% CI 1.09–2.40) compared to compensated DM2 individuals and 1.47 (95% CI 1.12–1.92) in relation to non-DM2 individuals. The apparent higher BMD detected in DM2 in our study is questionable since bone densitometry evaluates only the trabecular bone, which does not identify micro cracks of the cortical bone that occur in diabetic individuals. Therefore, it does not reflect impaired bone repair [61].

Severely obese women with sufficient vitamin D intake had higher total vertebral column BMD. This is in line with the findings of a recent meta-analysis of adults and older adults showing that vitamin D intake reduces the risk of total and hip fractures by 15% and 30%, respectively, due to bone fragility [62]. Vitamin D is essential for the promotion of calcium absorption, which favors proper bone mineralization, decreasing the risk of fractures [62,63]. Therefore, the ingestion and serum vitamin D level in severely obese patients requires attention, as 97.4% of pre-bariatric surgery obese women had moderate to severe vitamin D deficiency [64].

Our analyses found that insufficient serum levels of zinc were associated with lower vertebral column total BMD, similar to the finding in a study of Sadeghi et al. [65] showing that serum zinc was negatively correlated with femur T-score in women with osteoporosis. An in vitro study reported that zinc may stimulate osteoblastic cell proliferation, alkaline phosphatase activity (ALP), and bone matrix collagen synthesis [66]. In addition, zinc reduces bone reabsorption by inhibiting the formation of osteoclasts from bone marrow cells and promoting cellular apoptosis of mature osteoclasts [67]. In addition, 73.9% of obese women candidates for bariatric surgery had low serum zinc [68].

In the present study, elevated CRP levels were associated with lower total vertebral column BMD. CRP is an important inflammatory marker, which reinforces the finding of this study that the higher degree of obesity compromises bone health due mainly to low-grade inflammation [68,69,70]. Findings from the National Health and Nutrition Examination Survey (NHANES 1999–2004) in the adult and older adult American population identified that total body, subtotal, extremities and trunk BMD were inversely associated with CRP quintiles in both sexes, regardless of comorbidities, medications and serum levels of vitamin D [71]. 

The association of BMI ≥ 50 kg/m^2^ with lower total vertebral column and hip BMD is relevant. It is particularly relevant because there is a hypothesis that the mechanical overload of obesity can increase bone mass [7]. Our findings corroborate with the physiological pathway in which inflammatory and endocrine factors present in obesity and, more specifically, related to visceral adipose tissue and higher percentage of fat, can compromise the quality of bone mass [2,3,13,44,68,69,72]. A parallel comparison can be made with a study that identified that 68% of women with BMI ≥ 40 kg/m^2^ had the lowest quintile of femoral neck BMD [13]. Unlike our study, Maïmoun et al. [10] observed that Z scores were higher than normal values, with this difference being more evident for total body, lumbar 1–4 and hips in those women with BMI > 40 kg/m^2^, and also emphasized that lean mass, but not fat, was independently associated with BMD in men and women. Our results are also supported by a meta-analysis of adult and older women that found that a BMI increase of 1 kg/m^2^ above 25 kg/m^2^ corresponded to a 1% increase in fracture risk [73]. 

The risk for lower total BMD in individuals with BMI greater than 50 kg/m^2^ has generated an important discussion, since initially it was thought that higher BMI was linked to greater BMD, based on previous studies [7,8,10,54,61] on bone microarchitecture in obese people. Sukumar et al. [74] evidenced that severe obesity increased trabecular BMD and in the presence of a higher PTH was associated with a lower cortical BMD without prejudicing bone geometry and strength. Our result is also in agreement with a cohort that analyzed various bone sites, and showed that women who had lower bone strength in relation to body load had a higher risk of fracture and increased impact at the time of the fall [75]. The association of BMI with low BMD is more complex and seems to be different depending on the bone site analyzed [2,9,10,73,76].

As a potential limitation of this study, we could mention recall bias related to the food consumption data. In order to minimize these self-reporting errors, three 24 HR assessments were collected to calculate the mean intake of calcium and vitamin D. The results have internal and external validity due to the diverse methodological care adopted throughout the research carried out exclusively with severely obese individuals.

## 5. Conclusions

In conclusion, this study showed that BMI ≥ 50 kg/m^2^ is a risk factor for lower BMD. In addition, some common bone health risk factors that frequently occur in non-obese subjects also occurred in severely obese patients, including age ≥ 50 years, menopause, previous fracture, smoking, high CRP levels and low serum zinc levels. The protective factors for BMD were physical activity, DM2 and adequate vitamin D intake. Finally, some associations in specific bone sites were observed, which reinforce the need for further studies in order to clarify this condition and enlarge knowledge in this field.

## Figures and Tables

**Table 1:** Biochemical tests, methods and reference values.

Test	Method	Normal Values	Reference
Calcium	Endpoint colorimetric–Arsenazo III	8.5 to 10.5 mg/dL	Peacock [32]
25 Hydroxyvitamin D	Electrochemiluminescence	≥30 ng/dL	Maeda et al. [33]
PTH	Electrochemiluminescence	15–65 pg/mL	Marcocci, Cetani [34]
Zinc	Atomic absorption spectrophotometry	70–120 µg/dL	Yanagisawa [35]
HOMA-IR	Electrochemiluminescence	≤2.71	Geloneze et al. [36]
Fasting insulin	Electrochemiluminescence	2.6–24.9 µU/mL	Matthews et al. [37]
Fasting glucose	Enzymatic colorimetric	<100 mg/dL	ADA [30]
Glycated Hb	Immunoturbidimetry	<6.5%	ADA [30]
Free T4	Electrochemiluminescence	0.7–1.8 ng/dL	Chopra [38]
TSH	Electrochemiluminescence	0.45–4.12 mUI/L	Garber [31]
CRP (mg/L)	Immunochemical agglutination reaction	<6 or non-reactive	PCRTEST-Doles [39]

PTH (parathyroid hormone); HOMA-IR (homeostatic model assessment of insulin resistance); Hb (hemoglobin); T4 (tetraiodothyronine); TSH (thyroid stimulating hormone); CRP (C-reactive protein); ADA (American Diabetes Association).

**Table 2:** Associations between three bone mineral density (BMD) sites with sociodemographic variables, lifestyle and clinical conditions in severely obese women, DieTBra Trial, 2016 (*n* = 104).

Variables	Total *n* (%)	BMD Total Body (g/cm^2^) Mean ± SD	*p*	BMD Vertebral Column Total (g/cm^2^) Mean ± SD	*p*	BMD Hip Total (g/cm^2^) Mean ± SD	*p*
Age (yeas)			0.000 *		0.000 *		0.000 *
18–39	49 (47.1)	1.078 ± 0.095 ^a^		1.062 ± 0.151 ^a^		1.218 ± 0.107 ^a^	
40–49	40 (38.5)	1.318 ± 0.088 ^b^		1.113 ± 0.155 ^b^		1.216 ± 0.126 ^b^	
≥50	15 (14.4)	1.206 ± 0.053 ^ab^		0.927 ± 0.124 ^ab^		1.063 ± 0.165 ^ab^	
Skin Color			0.803 **		0.126 **		0.825 **
White	88 (84.6)	1.284 ± 0.095		1.052 ± 0.157		1.196 ± 0.134	
Black	16 (15.4)	1.278 ± 0.097		1.118 ± 0.168		1.188 ± 0.141	
Education (years)			0.391 ***		0.117 ***		0.749 ***
<9	34 (32.7)	1.272 ± 0.101		1.027 ± 0.159		1.189 ± 0.144	
≥9	70 (67.3)	1.289 ± 0.091		1.0792 ± 0.158		1.198 ± 0.129	
Socioeconomic class			0.231 **		0.906 **		0.274 **
A, B and C	86 (82.7)	1.278 ± 0.091		1.061 ± 0.156		1.188 ± 0.137	
D and E	18 (17.3)	1.307 ± 0.107		1.066 ± 0.178		1.227 ± 0.108	
Smoking			0.043 **		0.103 **		0.027 **
Never smoked	73 (70.2)	1.295 ± 0.093		1.078 ± 0.154		1.214 ± 0.129	
Ex-smoker/ Smoker	31 (29.8)	1.254 ± 0.092		1.023 ± 0.168		1.151 ± 0.137	
Binge drinking (*n* = 59)			0.939 **		0.428 **		0.115 **
Yes	31 (52.5)	1.292 ± 0.096		1.083 ± 0.155		1.229 ± 0.098	
No	28 (47.5)	1.290 ± 0.090		1.049 ± 0.173		1.173 ± 0.166	
Solar exposure			0.398 **		0.321 **		0.828 **
Yes	74 (71.1)	1.288 ± 0.095		1.072 ± 0.169		1.193 ± 0.133	
No	30 (28.9)	1.271 ± 0.094		1.037 ± 0.132		1.199 ± 0.138	
Solar time min/day (*n* = 74)			0.507 **		0.770 **		0.965 **
<20 min	14 (18.9)	1.303 ± 0.088		1.060 ± 0.189		1.192 ± 0.111	
≥20 min	60 (81.1)	1.284 ± 0.097		1.075 ± 0.166		1.194 ± 0.139	
PA ≥ 150min/week (*n* = 98)			0.385 **		0.132 **		0.064 **
Yes	4 (4.1)	1.324 ± 0.091		1.182 ± 0.191		1.316 ± 0.042	
No	94 (95.9)	1.282 ± 0.094		1.059 ± 0.158		1.188 ± 0.135	
Prior fracture (*n* = 92)			0.114 **		0.005 **		0.129 **
Yes	25 (27.2)	1.256 ± 0.087		0.986 ± 0.147		1.157 ± 0.157	
No	67 (72.8)	1.292 ± 0.099		1.086 ± 0.151		1.205 ± 0.125	
Menopause (*n* = 104)			0.025 **		0.025 **		0.000 **
Yes	18 (17.3)	1.238 ± 0.089		0.986 ± 0.201		1.092 ± 0.166	
No	86 (82.7)	1.292 ± 0.093		1.078 ± 0.146		1.217 ± 0.117	
Diabetes Mellitus 2			0.039 **		0.244 **		0.722 **
Yes	21 (20.2)	1.321 ± 0.090		1.026 ± 0.201		1.204 ± 0.142	
No	83 (79.8)	1.273 ± 0.094		1.071 ± 0.147		1.193 ± 0.133	
Hypothyroidism			0.182 **		0.309 **		0.448 **
Yes	18 (17.3)	1.256 ± 0.095		1.027 ± 0.177		1.173 ± 0.165	
No	86 (82.7)	1.289 ± 0.094		1.069 ± 0.156		1.199 ± 0.127	
Medication ↓BMD			0.062 **		0.702 **		0.620 **
Yes	27 (26.0)	1.254 ± 0.106		1.052 ± 0.175		1.184 ± 0.170	
No	77 (74.0)	1.293 ± 0.089		1.066 ± 0.155		1.199 ± 0.122	
BMI (kg/m^2^)			0.528 *		0.046 *		0.104 *
35–39.9	20 (19.3)	1.263 ± 0.079		1.110 ± 0.133 ^a^		1.221 ± 0.126	
40–49.9	73 (70.2)	1.286 ± 0.098		1.064 ± 0.163		1.199 ± 0.135	
≥50	11 (10.6)	1.298 ± 0.096		0.963 ± 0.147 ^a^		1.117 ± 0.121	
Calcium intake (mg/dia)			0.150 **		0.879 **		0.382 **
Adequate	5 (4.8)	1.343 ± 0.088		1.073 ± 0.145		1.247 ± 0.118	
Insufficient	99 (95.2)	1.280 ± 0.094		1.061 ± 0.161		1.192 ± 0.135	
Vitamin D intake (UI/dia)			0.639 **		0.119 **		0.510 **
Adequate	4 (3.8)	1.305 ± 0.106		1.184 ± 0.134		1.239 ± 0.092	
Insufficient	100 (96.2)	1.282 ± 0.094		1.057 ± 0.159		1.193 ± 0.136	

* ANOVA; ** Student’s T; *** Kruskal–Wallis test; ^a, b^ equal letters = different means; BMD (bone mineral density); SD (standard deviation); BMI (body mass index); Min (minutes); PA (physical activity); Bold font: statistically significant.

**Table 3:** Associations between bone mineral density in three sites and biochemical tests in severely obese women, DieTBra Trial, 2016 (*n* = 104).

Variables	Total *n* (%)	BMD Total Body (g/cm^2^) Mean ± SD	*p* *	BMD Vertebral Column (g/cm^2^) Mean ± SD	*p* *	BMD Hip (g/cm^2^) Mean ± SD	*p* *
Fasting glucose (mg/dL)			0.063		0.687		0.799
≤99	62 (59.6)	1.269 ± 0.089		1.057 ± 0.162		1.198 ± 0.139	
≥100	42 (40.4)	1.304 ± 0.099		1.069 ± 0.157		1.191 ± 0.127	
Glycated HB%			0.784		0.358		0.969
Normal	68 (65.4)	1.281 ± 0.093		1.073 ± 0.141		1.195 ± 0.128	
Elevated	36 (34.6)	1.287 ± 0.098		1.042 ± 0.189		1.194 ± 0.147	
Homa-IR			0.787		0.481		0.940
Normal	16 (15.4)	1.277 ± 0.084		1.088 ± 0.120		1.193 ± 0.099	
Elevated	88 (84.6)	1.284 ± 0.097		1.057 ± 0.166		1.195 ± 0.140	
Fasting insulin (uU/mL)			0.915		0.344		0.204
Normal	76 (73.1)	1.284 ± 0.09		1.053 ± 0.157		1.185 ± 0.141	
Elevated	28 (26.9)	1.281 ± 0.102		1.087 ± 0.167		1.222 ± 0.110	
CRP (mg/L)			0.841		**0.040**		0.139
Normal	41 (39.4)	1.285 ± 0.109		1.101 ± 0.148		1.219 ± 0.120	
Elevated	63 (60.6)	1.281 ± 0.085		1.036 ± 0.163		1.179 ± 0.141	
PTH (pg/mL)			0.548		0.129		0.659
Normal	15 (14.4)	1.296 ± 0.076		1.120 ± 0.148		1.209 ± 0.114	
Elevated	89 (85.6)	1.281 ± 0.097		1.052 ± 0.160		1.193 ± 0.138	
Free T4 (ng/dL)			0.262		0.349		0.508
Normal	102(98.1)	1.284 ± 0.095		1.064 ± 0.159		1.196 ± 0.135	
Elevated	2 (1.9)	1.208 ± 0.032		0.957 ± 0.182		1.132 ± 0.036	
TSH (mUI/L) (*n* = 103)			0.327		0.823		0.072
Normal	86 (83.5)	1.288 ± 0.093		1.066 ± 0.156		1.207 ± 0.132	
Elevated	17 (16.5)	1.263 ± 0.101		1.056 ± 0.174		1.142 ± 0.136	
Serum calcium (mg/dL)			0.261		0.583		0.855
Normal	95 (91.4)	1.286 ± 0.095		1.065 ± 0.162		1.194 ± 0.136	
Reduced/elevated	9 (8.6)	1.249 ± 0.089		1.034 ± 0.142		1.203 ± 0.117	
Serum Vitamin D (ng/mL)			0.874		0.596		**0.043**
Normal (>30)	53 (50.9)	1.284 ± 0.104		1.070 ± 0.167		1.169 ± 0.142	
Insufficient (<30)	51 (49.1)	1.281 ± 0.085		1.053 ± 0.152		1.222 ± 0.121	
Serum Vitamina D (ng/mL)			0.799		0.324		0.106
Normal (>20)	92 (88.5)	1.284 ± 0.095		1.068 ± 0.159		1.203 ± 0.121	
Insufficient (<20)	12 (11.5)	1.276 ± 0.092		1.019 ± 0.168		1.136 ± 0.209	
Serum Zinc (µg/dL)			0.882		**0.016**		0.185
Normal	92 (88.5)	1.282 ± 0.096		1.075 ± 0.156		1.201 ± 0.133	
Insufficient	12 (11.5)	1.287 ± 0.083		0.958 ± 0.152		1.147 ± 0.137	

* Student’s T; BMD (bone mineral density); SD (standard deviation); Hb (hemoglobin); CRP (C-reactive protein); PTH (parathyroid hormone); T4 (tetraiodothyronine); TSH (thyroid stimulating hormone); Bold font: statistically significant.

**Table 4:** Simple linear regression of three bone mineral density sites and their relationship with sociodemographic variables, health conditions, nutritional indicators and biochemical tests in severely obese women, DieTBra Trial, 2016 (*n* = 104).

Variables	BMD Total Body (g/cm^2^)		BMD Vertebral Column (g/cm^2^)		BMD Hip (g/cm^2^)	
	β	*p*	β	*p*	β	*p*
Age (years)		**0.000**		**0.000**		**0.000**
18–39	1.00		1.00		1.00	
40–49	0.040	**0.045**	0.051	0.113	−0.002	0.947
≥50	−0.072	**0.000**	−0.135	**0.003**	−0.155	**0.000**
Skin colour (black)	−0.006	0.803	0.066	0.126	−0.008	0.825
Education (≥9 years)	0.017	0.391	0.052	0.117	0.009	0.749
Economic class (D-E)	0.029	0.231	0.005	0.906	0.038	0.274
Smoking (ex-smoker/Smoker)	−0.041	**0.043**	−0.056	0.103	−0.063	**0.027**
Binge drinking (yes)	0.002	0.940	0.034	0.428	0.056	0.116
Solar exposure (yes)	0.017	0.399	0.034	0.321	−0.006	0.828
≥20 min sun/day (yes)	−0.0129	0.507	0.015	0.770	0.002	0.965
PA ≥150/week (yes)	0.042	0.385	0.123	0.132	0.127	0.064
Prior fracture (yes)	−0.036	0.114	−0.100	**0.005**	−0.048	0.129
Menopause (yes)	−0.055	**0.025**	−0.092	0.025	−0.124	**0.000**
Diabetes Mellitus (yes)	0.048	**0.039**	−0.045	0.244	0.012	0.722
Hypothyroidism (yes)	−0.033	0.183	−0.042	0.309	−0.026	0.448
Medicaments ↓BMD (yes)	−0.039	0.062	−0.014	0.702	−0.014	0.620
BMI (kg/m^2^)		0.528		**0.046**		0.104
35–39.9	1.00		1.00		1.00	
40–49.9	0.023	0.326	−0.047	0.240	−0.021	0.532
≥50	0.035	0.322	−0.148	0.013	−0.103	**0.040**
Calcium intake (mg/day) (adequate)	0.062	0.150	0.011	0.879	0.054	0.382
Vitamin D intake (UI/day) (adequate)	0.023	0.639	0.127	0.119	0.045	0.510
Serum calcium (mg/dL) (normal)	0.037	0.261	0.031	0.583	−0.009	0.855
Serum vitamin D (ng/mL) (insufficient) <20)	0.007	0.799	0.049	0.324	0.067	0.106
Serum zinc (µg/dL) (insufficient)	0.004	0.883	−0.117	**0.016**	−0.055	0.185
PTH (mUI/L) (elevated)	−0.016	0.549	−0.068	0.129	−0.017	0.659
Homa-IR (elevated)	0.007	0.787	−0.031	0.481	0.003	0.940
Fasting insulin (uU/mL) (elevated)	0.007	0.787	−0.031	0.481	0.003	0.940
Fasting glucose (mg/dL) (≥100mg/dL)	0.035	0.064	0.013	0.687	−0.007	0.799
Glycosylated HB (%) (elevated)	0.005	0.784	−0.030	0.358	−0.001	0.970
Free T4 (ng/dL) (elevated)	−0.076	0.262	−0.107	0.350	−0.064	0.508
TSH (mUI/L) (elevated)	−0.025	0.327	−0.009	0.823	−0.064	0.072
PCR (mg/L) (elevated)	−0.004	0.841	−0.065	**0.040**	−0.039	0.139

BMD (bone mineral density); Hb (hemoglobin); BMI (body mass index); Min (minutes); MVPA (moderate to vigorous physical activity); PTH (parathyroid hormone); T4 (tetraiodothyronine); TSH (thyroid stimulating hormone); Bold font: statistically significant.

**Table 5:** Multiple hierarchical linear regression between total body, vertebral column and hip bone mineral density adjusted for sociodemographic variables, health conditions, nutritional indicators and biochemical tests in severely obese women, Goiânia, DieTBra Trial, 2016 (*n* =104).

Variables	BMD Total Body (g/cm^2^)		BMD Vertebral Column (g/cm^2^)		BMD Hip (g/cm^2^)	
	β (CI 95%)	*p*	β (CI 95%)	*p*	β (CI 95%)	*p*
**1st level**						
Age (yeas)						
18–39	1.00		1.00		1.00	
40 a 49	0.039 (0.000; 0.079)	**0.045**	0 0.059(−0.003; 0.121)	0.060	−0.002 (−0.052; 0.048)	0.945
≥50	−0.072 (−0.109; −0.034)	**0.000**	−0.117 (−0.195; −0.040)	**0.000**	−0.155 (−0.243; −0.067)	**0.001**
Skin color						
White/Brown	−		1.00		−	
Black	−		0.054 (−0.029; 0.138)	**0.203**	−	
Education (years) **						
<9	−		1.00		−	
≥9	−		0.051 (−0.008; 0.110)	**0.092**	−	
**2nd level**						
Smoking						
Never smoked	1.00		1.00		1.00	
Ex-smoker/ Smoker	−0.040 (−0.080; −0.001)	**0.045**	−0.002 (−0.066; 0.061)	0.942	−0.007 (−0.079; 0.065)	0.853
Binge drinking (*n* =59)						
Yes	−		−		1.00	0.527
No	−		−		0.024 (−0.052; 0.100)	
PA ≥ 150min/week (*n* =98)						
Yes	−		1.00		1.00	
No	−		0.136 (−0.009; 0.283)	0.066	0.096 (0.039; 0.153)	**0.001**
Prior fracture (*n* =92)						
Yes	1.00		1.00		1.00	
No	−0.014 (−0.061; 0.033)	0.560	−0.085 (−0.146; −0.023)	**0.007**	−0.033 (−0.094; 0.027)	0.273
Menopause (*n* =104)						
Yes	1.00		1.00		1.00	
No	−0.022 (−0.088; 0.044)	0.514	0.025 (−0.156; 0.206)	0.787	−0.147 (−0.231; −0.063)	**0.001**
Diabetes Mellitus 2						
Yes	1.00		−		−	
No	0.082 (0.037; 0.126)	**0.000**			−	
**Cont.**	**BMD Total Body (g/cm^2^)**		**BMD Vertebral Column (g/cm^2^)**		**DMO Hip (g/cm^2^)**	
	**β (CI 95%)**	***p***	**β (CI 95%)**	***p***	**β (CI 95%)**	***p***
Hypothyroidism						
Yes	1.00		−		−	
No	−0.017 (−0.077; 0.043)	0.576	−		−	
Medication ↓BMD						
Yes	1.00					
No	−0.020 (−0.076; 0.036)	0.477				
BMI (kg/m^2^)						
35−39.9	−		1.00		1.00	
40−49.9	−		−0.023 (−0.094; 0.048)	0.525	−0.056 (−0.118; 0.005)	0.072
≥50	−		−0.133 (−0.246; −0.019)	**0.022**	−0.157 (−0.311−0.004)	**0.045**
Calcium intake (mg/day)						
Adequate	1.00		−		−	
Insufficient	0.024 (−0.070; 0.118)	0.616	−		−	
Vitamin D intake (UI/day)						
Adequate	−		1.00		−	
Insufficient	−		0.071 (0.004; 0.139)	**0.039**	−	
**3rd level**						
CRP (mg/L)						
Normal	−		1.00		1.00	
Elevated	−		−0.053 (−0.105; −0.000)	**0.049**	−0.012 (−0.061; 0.036)	0.613
Serum Zinc (µg/dL)						
Normal	−		1.00		1.00	
Insufficient	−		−0.120 (−0.19; −0.026)	**0.010**	−0.051 (−0.134; 0.032)	0.223
PTH (pg/mL)						
Normal	−		1.00		−	
Elevated	−		−0.004 (−0.085; 0.076)	0.918	−	
TSH (mUI/L) (*n* =103)						
Normal	−		−		1.00	
Elevated	−		−		−0.028 (−0.107; 0.051)	0.485
Serum Vitamin D (ng/mL)						
Normal (>30)	−		−		1.00	
Insufficient (<30)	−		−		0.023 (−0.031; 0.077)	0.394
Fasting glucose (mg/dL)						
≤99	1.00		−		−	
≥100	0.019 (−0.018; 0.056)	0.302	−		−	

BMD (bone mineral density); BMI (body mass index); MVPA (moderate to vigorous physical activity); CRP (C-reactive protein); Bold font: statistically significant.
